# In reply

**DOI:** 10.1093/oncolo/oyag110

**Published:** 2026-04-06

**Authors:** Emily R Mackler, Siobhan Norman, Farah Jalloul-Rizk

**Affiliations:** YesRx and University of Michigan College of Pharmacy, Ann Arbor, MI 48104, United States; YesRx, Ann Arbor, MI 48104, United States; Michigan Pharmacist Association, Lansing, MI 48933, United States

We thank Dr. Xu and colleagues for their letter titled “Low opinion of generic oral anticancer drugs: Unacknowledged products resulting in medication waste in China,” in response to our publication, “Statewide Cancer Drug Repository Reducing Waste in the Setting of Shortage Crisis,”^1^ as they raise important considerations for ongoing efforts to decrease pharmaceutical waste and improve medication access. The authors present two main reasons for oral anticancer medication waste in China—product size and public opinion of generic medications. As it relates to the ability to minimize waste and take advantage of cancer drug repository legislation, we see that product size and dosing availability are also problems in the United States. Medications provided in blister packs are more feasible for repurposing and help to minimize the medication waste resulting from discontinuations and dosage changes during treatment.

The low opinion of generic medications presented by the authors is a unique perspective that we may not have had the opportunity to fully assess in the United States, as most oral anticancer medications remain on patent. Generic drugs became available in the US after the Hatch-Waxman Act of 1984, allowing the FDA to approve generics via an Abbreviated New Drug Application, focusing on bioequivalence.^2^ While in most cases, generic drugs result in cost savings, reductions in cost for anticancer medications have been delayed.^3^ Furthermore, the cost of cancer drugs coming to market in China is nearly half that of the US,^4^ potentially creating a different patient perspective when generics are available. In a study assessing medication adherence between generic and brand imatinib for patients with chronic myeloid leukemia, researchers found a larger proportion of patients who took the generic product were adherent and persistent to therapy than those who took the brand, presumably due to lower out-of-pocket costs.^5^ We agree with the authors’ proposal that the government set appropriate drug prices and believe the same strategy would be helpful in the US—particularly since the majority of medication shortages occur within the generic drug market, where greater incentives are likely needed to improve the reliability of the manufacturer supply chain.^6^

## Conflicts of interest

None declared.
